# Risk factors for Extended Spectrum Beta-Lactamase (ESBL)- producing bacteria infections in ICU of Jogja Hospital

**DOI:** 10.1017/ash.2025.139

**Published:** 2025-09-03

**Authors:** Kian Sinanjung, Fatrinawati, Istiqomah, Gita Mayasari

**Affiliations:** 1 Clinical Microbiology; 2 Clinical Pathology; 3 Infection Prevention and Control Nurse; 4 Clinical Pharmacy

**Keywords:** Risk factor, ESBL infection, ICU

## Abstract

**Introduction:** Infection by Extended Spectrum Beta-Lactamase (ESBL)-producing bacteria in Intensive Care Unit (ICU) is associated with treatment failure, prolonged hospital stay, increased costs and patient mortality. The factors of infection by ESBL-producing bacteria are important to figure out so that prevention and control efforts can be made. The aim of the study is to understand the risk factors associated with ESBL- producing bacteria infections in ICU of Jogja Hospital. **Method:** This case control study included ICU’s patients who have an infection with confirmed *E. coli* or *K. pneumoniae* based on microbiological examination from January-December 2023. The patient’s data were obtained from the medical record to find the risks factors. Cases were defined as *E. coli* or *K. pneumoniae* ESBL, while controls were defined as non ESBL *E. coli* or *K. pneumoniae*. Bivariate analysis was carried out on independent variables (sex, age, length of hospitalization, comorbidity, history of cephalosporin, duration of antibiotic, utilization of urinary catheter and central venous catheter, exposure to mechanical ventilation, and presence of open wounds). Risk factors with P values <0.25 on bivariate analysis were included in multivariate logistic regression analysis. Two- sided P values less than 0,05 were considered statistically significant. **Result:** There were 30 patients with ESBL-producing *E. coli* or *K. pneumoniae* (cases) and 32 patients with non ESBL-producing *E. coli* or *K. pneumoniae* (controls). Based on multivariate analysis, the presence of open wounds has statistical significance, OR 6.52 (IK 95% p = 0.011). **Conclusion:** The presence of open wounds is associated with the occurrence ESBL-producing *E. coli* or *K. pneumoniae* infection in ICU of Jogja Hospital.

